# The experience of college students with pulmonary tuberculosis in Shaanxi, China: a qualitative study

**DOI:** 10.1186/1471-2334-10-174

**Published:** 2010-06-16

**Authors:** Shao-Ru Zhang, Hong Yan, Jin-Jing Zhang, Tian-Hua Zhang, Xiao-Hong Li, Yin-Ping Zhang

**Affiliations:** 1 Department of Nursing, College of Medicine, Xi'an Jiaotong University, Xi'an city, China; 2 Department of Public Health, College of Medicine, Xi'an Jiaotong University, Xi'an city, China; 3 Shaanxi Provincial Institute for Tuberculosis Control and Prevention, Xi'an city, China

## Abstract

**Background:**

The prevalence of pulmonary tuberculosis among college students in Shaanxi is high. Although tuberculosis leaves much psychological and social impact on patients, little is known about its impact on college students. The objective of this study is to explore the experiences and psychological process of college students with pulmonary tuberculosis in Shaanxi, China.

**Methods:**

17 college students with pulmonary tuberculosis were recruited purposively from 9 colleges in Shaanxi. In-depth interviews were conducted to collect data and a thematic framework analysis was used.

**Results:**

The participants reported that pulmonary tuberculosis deeply influenced their mental health. They were fearful to the nature of pulmonary tuberculosis at the stage of diagnosis, anxious about the illness before the period of diagnosis and the early week of the treatment, excessive worry immediately before the first recheck. They expected an early full recovery, bored on tedious treatment life and worried about future heath and prospects during the whole treatment phase. Their daily life was also influenced, namely discontinued studies, isolation and increased financial burden. They also reported that they could get strong supports from family members, while little supports from healthcare workers and their friends.

**Conclusions:**

The participants' psychological pressure was significant during the treatment. In addition, there was serious conflict between treatment and study; social support provided for them was insufficient. Healthcare workers should provide psychological support for college students with pulmonary tuberculosis according to the psychological characteristics and offer social support through strengthening communication with them. Colleges should follow governmental policies on TB exactly and provide opportunities for the patients to continue their studies.

## Background

The prevalence of pulmonary tuberculosis (PTB) among college students is high in China [1]. College students are usually 18-23 years old, with the nature of rapid growth, development and endocrine instability. It has been reported that incidence of PTB started to rise greatly at this period [2]. In China, there are more than 25 million college students studying in 2,311 colleges to date. Although the number of colleges has almost doubled and the number of undergraduates has increased by six-fold in the past 10 years, the infrastructure of the colleges could not keep pace with the expansion [3]. Up to now, 6 to 8 people live in one dormitory room, 60 to 200 students attend lectures in one classroom, and 1000 to 2000 people dine in the same hall. Densely dwelling, close contact are salient features of the Chinese colleges [4]. In recent years, there were several reported PTB epidemics in Chinese colleges [5,6]. In Shaanxi province, which has the third largest number of colleges in China, there are 847.2 thousand college students in 76 colleges [7]. Shaanxi Provincial Institute for Tuberculosis (TB) Control and Prevention has unpublished data that in Shaanxi in 2005, students accounted for 12.5% of 39,822 PTB cases reported. Only farmers accounted for more. College student cases accounted for 21.8% of student cases. PTB incidence in college students in Shaanxi was 143.1/100,000, significantly higher than 108.5/100,000, the provincial average.

It is reported that TB caused plenty problems for patients, including stigmatization and social isolation of TB patients and their families, diminished marriage prospects for young TB patients and their family members, even the divorce of the married [8,9]. Diagnosis of TB also leads to depression and anxiety [10]. Most Chinese college students with PTB experience negative emotion, including anxiety, moping, tension, pessimism, etc. [11]. A better understanding of the experiences of college students with PTB is useful for making a comprehensive plan of psychological and social support for college students with PTB. However, there are few studies reported on it. Using a qualitative approach, this study explored the experiences and psychological process of college students with PTB.

## Methods

### Study design

The study design was qualitative, using an in-depth interview approach.

### Study setting

Shaanxi province was conveniently selected. The province is located in the north western part of China, consists of 10 cities including 107 districts and counties. It is a less-developed inland province. There are 76 colleges in Shaanxi. 56 colleges accounting for 74% located in Xi'an city, the provincial capital. It is reported that the monthly consumption level of a college student in Shaanxi ranges from RBM 100 to RMB 3000 with an average of RMB 461.3. In 2008, 80% college students in Shaanxi spent about RMB 6,000 to cover their one year expense [12]. Most of college students come from countryside and annual capita income of China's rural family was RMB 5,153 [13].

There is a TB department at every Centre for Disease Control (CDC) at district level and a TB clinic in designated hospital. In 2003, 'The notice on strengthening PTB control and prevention work in schools' was published by the Ministry of Health and Ministry of Education of The People's Republic of China. In this document, college students with PTB are required to be centrally managed. According to the policy of convergence management, which was progressively implemented from 1992 in China, all health providers including college clinics, general hospitals, and professional TB hospitals where a college student might be diagnosed with PTB must report the case through the TB surveillance network and refer the student to a district CDC or a designated hospital. The TB department at district CDC and TB clinic in designated hospital where TB free treatment policy is implemented are responsible for TB diagnose and treatment. TB free treatment policy covers costs of the whole course of first line anti-TB drugs, TB sputum smears and cultures, and X-ray examinations. Other fees, such as hospitalization fees, any other drugs and medical examinations, are not covered by the policy.

After a college student is diagnosed with PTB, some of the colleges will inform the student that he or she can receive free anti-TB drugs from CDC or the designated hospital without being hospitalized if he or she needs not. Usually, students in these colleges prefer to receive free drugs. However, most of the colleges will directly refer the students to TB hospitals, where the students are not covered by free treatment policy, and most of the students in those colleges accept it. Treatment of active PTB lasts at least 6 months. A patient who is hospitalized can be discharged at the end of intensive phase (the first 2 months). Response to treatment is monitored at the end of the second, fifty and sixth month of the therapy phase. A patient whose sputum smear is positive at the end of second month should be rechecked at the end of third month. However, inpatients and outpatients are also rechecked practically at the end of the first treatment month prescribed by their health providers.

### Participants

This study was approved by the University Ethics Committee. The participants verbally agreed to be interviewed or signed informed consent. The principle of data saturation, which means no new information being found, was used to determine the number of participants. After each interview, preliminary analysis of the data was conducted so that the next participant who would supplement maximum information could be recruited. We halted the recruitment process when there was an indication of information saturation.

With a purposive sampling method applying the maximum variation technique, 17 college students diagnosed and recorded as PTB patients by district CDC or designated hospital participated in the study. Participants were selected according to school, type of degree and year of study in order to represent college students with PTB in Shaanxi province. The researchers collected the records of all college students with PTB from Shaanxi Provincial Institute for TB Control and Prevention to list the name of colleges with PTB patients, and then proportionally chose 6 colleges in Xi'an city and 3 colleges not affiliated to Xi'an city. Doctors in the 9 colleges were asked to contact the students with PTB in their colleges respectively. The college doctors provided the contact information including the patient's phone number to the interviewers, once the patient volunteered to be interviewed. The study was planned to balance the number of undergraduates and postgraduates (nearly 12:1 in Shaanxi), unfortunately only one master degree student and one PhD student agreed to participate in. Meanwhile, we balanced the undergraduates according to the year of study (3 freshmen, 4 sophomores, 4 juniors, 4 seniors). According to the aim of the study, the researcher selected patients in different treatment phase, including intensive phase (the first 2 months) and continuation phase (the remaining 4 months), in order to get saturated information of their experience. 11 of the 17 paticipants were in intensive phase when the interview was conducted, and the others were in continuation phase. One of the 11 patients who were in intensive phase was not hospitalized, therefore 10 of the 17 were inpatients. All of the participants were diagnosed with PTB for the first time and were smear-negative. The researchers failed to get in touch with the participants ascertained with smear-positive, recurrent PTB, or multi-drug resistant PTB. None of the participants majored in medicine.

### Data collection

The interview was mainly conducted by the principal researcher and assisted by postgraduates. The postgraduates were trained to collect data for qualitative research before the interviews. In September 2008, to ensure the effectiveness of the data collection and analysis, the researchers selected two cases to make a pilot interview. The raw data was transcribed into words, and the research team read and analyzed it together. The formal interviews were conducted from October to November 2008.

Inpatients were interviewed individually in a private office; outpatients were interviewed in a place convenient for both the interviewer and the participant. In-depth interview was used to collect data. The interview was voluntary and adhered to the principles of nondisclosure and convenience. The interviewer explained the aim and process of the study. The participants understood the necessity of recording and the nondisclosure of their private information. Two interviewers were included in each interview: one mainly responsible for interviewing, and the other primarily responsible for noting and audio-recording. The interviews were conducted in mandarin Chinese.

Fielding & Fielding's Triangle correction was used in the interview. Nonverbal information, such as tones, facial expressions, and gestures, was observed during the interview. An interview schedule was used to collect data (see Appendix 1), and the interviewer guided the participants in expressing their feeling, thinking, psychological process and experience during the illness. Each case was interviewed for 50-70 minutes. To accomplish this task, the interviewer set aside, as much as possible, any preconceived notions, expectations, or frameworks about the phenomenon and opened themselves fully to the process. All of this could enhance the accuracy of the study.

### Data analysis

Thematic framework analysis was used. Two researchers transcribed the audio-record word-by-word after each interview, and then listened to the audio-record and read noted observations to check accuracy of the transcript. After repeatedly read the transcripts, code frame was progressively established based on recurring viewpoints emerging from the data and the interview guideline. Every transcript was then coded systematically against the code frame. Codes were merged into categories and then these categories were organized into themes. Disagreements were discussed among the research team to reach a final consensus. The principal researcher revisited the main points of the findings with the participants and asked whether they were consistent with their experiences. Data analysis was conducted in Chinese and the final report was translated into English.

## Results

The 17 participants' demographic characteristics are summarized in Table 1.

**Table 1:** Demographic characteristics of the participants

	Number
Gender	
Male	12
Female	5
Age	
<20	2
20-30	14
>30	1
Type of degree	
Undergraduate	15
Master student	1
PhD student	1
Native place	
Countryside	13
Urban	4
Phase of treatment	
Intensive phase	11
Continuation phase	6
Time of receiving treatment	
<1 month	6
1-2 month	5
>2 month	6

Three main themes were generated after analysis, as follows:

### Influence on mental health

College students with PTB described various emotions related to their illness, such as anxiety before diagnosed with PTB, badly fear when diagnosed with PTB, anxiety in the beginning of treatment, excessive worry immediately before the first recheck, worry, expectancy and boredom during the whole treatment phase.

#### Fear about the nature of illness

The majority of the participants reported that their initial reaction to diagnosis of tuberculosis was quite fearfull. They said that they knew little about PTB before they were diagnosed. In their minds, PTB is a very serious disease that would badly harm them. Some of them considered PTB as a disease that could not be cured, and some even feared that they would die from PTB.

*'At the news that I was diagnosed with PTB, I was badly fearful. When I was a child, I often heard that if one suffered from it...he would die.' *(Male, 20 years old, continuation phase, outpatient)

Two participants reported that although they were not scared, it was hard for them to accept it when diagnosis of PTB was disclosed to them. '*How could I be suffered from PTB? It is absolutely unexpected for me.*' (Female, 18 years old, intensive phase, inpatient)

Some participants reported not only a great fear of the harms caused by PTB, but also the fear of infecting their classmates considering the infectious characteristic of PTB. One participant described his psychological process when his classmates were screened as intimate contactors.

*'It is an infectious disease. I was worry about the transmission. Whether my classmates and roommates would be infected by me? Oh, I was fearful, badly fearful when thought of it.' *(Male, 22 years old, continuation phase, outpatient)

The types of isolation intervention in TB hospitals indicated the patients that PTB was seriously infectious and it would greatly threaten their health, which deepened their feelings of fear.

*'I noticed that many people here wear big surgical masks and it seemed strange. I felt badly afraid at first.' *(Male, 21 years old, intensive phase, inpatient)

#### Anxiety about the illness

Most of the patients reported they felt anxious and upset before they were diagnosed. They repeatedly received symptomatic treatment, but could not be cured. They began to confuse the status of them, and they were upset and anxious.

*'I suddenly caught a cold and went to see a doctor. However, I caught colds at least three times even after careful medication. Oh, why is it so difficult for me to recover from catching a cold?' *(Male, 22 years old, intensive phase, outpatient)

*'I had a fever. I told myself that I just caught a cold and everything would be fine. However, it could not be cured for a long time. I began to feel upset--worse and worse.' *(Male, 21 years old, intensive phase, inpatient)

At the first week of receiving treatment, some of the patients still could not accept the fact that they suffered from PTB. Although some of the patients accepted it, they were troubled by the possible problems would caused by PTB, such as high expenditure and time-consuming treatment, and they could not fulfil themselves into receiving treatment.

*'I had no symptoms. At first, I thought if not suffering from PTB, I could be seriously infected during hospitalization by other PTB patients. What should I do? Those thoughts crammed my mind, and then I had no taste even for the delicious food. I could not sleep well.' *(Male, 22 years old, intensive phase, inpatient)

*'During the first week of hospitalization, I went to see other doctors to make sure that it really would take me at least 6 months to cure the disease. I went to bed early, but I could not sleep deeply and was easy to be woken up.' *(Male, 21 years old, continuation phase, outpatient)

#### Excessive worry about first recheck

All the participants who had received treatment more than one month reported they were tense and fearful when they will get a check-up after one-month treatment. They were eager to know whether the treatment for the past month was effective or not, but they were badly afraid of the bad news. The two thoughts were mixed and crashed in their minds.

*'I really expected to get a check again. However, when the day came, I was worried. If there was any problem, I would have to stay here for a longer time. I was extremely worried. This feeling lasted till I was checked again.' *(Male, 21 years old, intensive phase, inpatient)

*'It was time to be checked again. I was panic, it cannot be expressed in words. I was worry about the results, whether it is effective or not?' *(Male, 22 years old, intensive phase, outpatient)

#### Expect an early full recovery

Some patients reported that what they concerned most was the effect of the treatment. Inpatients expected that they could be healed and discharged from the hospital as soon as possible.

*'The doctor said that the treatment was effective. I could leave here in 10 days. Once I am cured thoroughly, I will not be worried.' *(Male, 22 years old, intensive phase, inpatient)

*'I just hope I could be cured thoroughly and get a full recovery, and then I can set my minds at rest.' *(Male, 24 years old, continuation phase, outpatient)

#### Worry about future heath and prospects

'*PTB will relapse if my study or work is stressful in the future. This problem bothered me a lot.*' said a student who would recover soon. (Male, 22 years old, continuation phase, outpatient)

Some patients were worried that the side effects of anti-TB drugs would exist for long terms, especially the outcome of infertility and impaired function of liver and kidney.

*'It is said that taking anti-TB drugs might lower fertility, I am rather worried about it.' *(Male, 20 years old, continuation phase, outpatient)

The seniors would graduate. Therefore, they were worried about that whether their graduation would be postponed since they were long time absence from class and job seeking opportunity would also be impacted.

*'Everything will be difficult for me, if I could not be cured. Maybe I will not be employed...' *(Male, 22 years old, intensive phase, inpatient)

#### Boredom on tedious treatment life

Inpatients only needed an intravenous infusion for a short time and took pills on a regular schedule every day. Outpatients who stayed at home also merely took medicine on time daily and none housework would be assigned to them. There was too much spare time for them, and they became bored.

*'I began to take a drip at 8 o'clock and it ended at 10 AM every morning. Then I had nothing to do the whole day...' *(Male, 22 years old, intensive phase, inpatient)

*'I have nothing to do at home, one month, another month. I am bored to death! After all, I am a young man.' *(Male, 21 years old, continuation phase, outpatient)

### Influence on daily life

#### Discontinuation of study

The biggest problem caused by PTB was the disruption of the participants' normal life, especially their studies when receiving treatment. All 17 participants expressed that they strongly desired to study during the treatment. Most of them were unwilling to suspend their schooling duo to the treatment.

*'My classmates took action to prepare for postgraduate study or going abroad. But, I am behind in my study and can not do anything. I am so worried.' *(Female, 22 years old, intensive phase, inpatient)

*'Since I was absence from school for a period, my teachers did not allow me to take exams. And I have to suspend my schooling. It will delay my education by one year!' *(Male, 22 years old, intensive phase, inpatient)

*'If suspending one's schooling is a rule in my school, I can accept it, rationally. However, once recover from PTB, I prefer to go back to school.' *(Male, 22 years old, intensive phase, outpatient)

#### Being isolated

College students with PTB were unwilling to disclose their disease, because they were afraid of being discriminated. Most of the participants also said that their classmates kept them away intentionally or unintentionally because they did not want to be infected.

*'I did not want to see anyone during the treatment. After supper, I sat in the yard for a while and then went to bed. My mother told others I was suffered from pleurisy. We did not tell them the truth, because most of the villagers like to gossip.' *(Male, 20 years old, continuation phase, outpatient)

*'My closest classmates were estranged from me...but I can understand (smile). If a classmate of mine suffered from PTB, maybe I would also do it.' *(Male, 21 years old, intensive phase, inpatient)

*'I am self-abased. Others may be disgusted with me. Although I am no longer a source of infection, I still keep away from others.' *(Male, 21 years old, intensive phase, inpatient)

#### Increased financial burden

Most of the participants came from rural areas, and long-term treatment cost them too much. Supplementary drugs used to reduce the side effects of anti-TB drugs were expensive, and improving notorious status also spent money. Therefore, PTB financially burdened some of the patients.

*'I come from rural area. Of course, my biggest problem is how to deal with the costs.' *(Male, 21 years old, intensive phase, inpatient)

*'There are 4 children in my family: 2 are in high school, and 2 are in college. My parents rely on farming and a part-time job to support us. Now, they have to pay so much for the disease.' *(Male, 24 years old, continuation phase, outpatient)

### Social support

#### Support from healthcare workers

The majority of the participants reported that they had little chance to communicate with healthcare workers. And they could get very little information on PTB from professionals. Most of the patients were unclear about what convergence management policy and TB free treatment policy were. Only 2 reported that doctors in their college clinics told them the two policies. The majority of the participants also reported that they knew little information about PTB before they were diagnosed with PTB. They got information by communicating with other patients, reading the posters in the hospital or browsing websites. However, they have not been offered any written information bulletin on PTB. Although most of them could state the symptoms, route of transmission, pathogen, duration of treatment after a period of treatment, some still had misconceptions about PTB.

*'I knew nothing about PTB before hospitalization. Although, I've been here for half a month, I still know little about it.' *(Female, 20 years old, intensive phase, inpatient)

*'The way I infected? Oh...maybe I ate something not clean.' (*Female, 18 years old, intensive phase, inpatient)

Most of the inpatients reported that the doctors and nurses in the hospital communicated with them occasionally, and they could get little information about their status from doctors and nurses.

*'When I asked questions, the doctor only told me to have a good rest. He even did not tell me any information about my illness.' *(Male, 24 years old, intensive phase, inpatient)

#### Support from family members

Most of the participants reported that their family members provided supports for them both physically and emotionally. When they were distressed, their parents or siblings would console them in time. Some inpatients reported that their parents came to look after them for a period from their hometown. Patients who went home to finish continuation phase treatment reported they were well taken care of by their family members.

*'My parents always told me not to be worried and they can help me. My elder sister also consoled me frequently.' *(Male, 21 years old, intensive phase, inpatient)

#### Support from friends

Some patients reported that some of their best friends were as friendly as before after they were diagnosed with PTB. Some of their friends even collected information on PTB for them.

*'After I was diagnosed with PTB, several best friends of mine searched information about PTB on the internets, and told me what food was good for my health. It made me feel well.' *(Male, 20 years old, continuation phase, outpatient).

During the treatment, their friends constantly encouraged them, which empowered them. One patient stated how one of his desk mates supported him.

*'She sent me a short message everyday, it was a joke or a phrase which can console me. Just like I was hurt, she cured me.' *(Male, 22 years old, intensive phase, outpatient)

However, most of the patients reported that they could not get any support from their friends, and some even kept away from them.

*'I have already gone back to school, some of my friends do not treat me as before, and I am feeling uncomfortable, even that I would like to quit school.' *(Male, 24 years old, continuation phase, outpatient)

## Discussion

This is the first qualitative study exploring the experiences of college students with PTB in the mainland of China. The target population included inpatients and outpatients. The use of in-depth interviews elicited rich and comprehensive information on the experience and psychological process of college students with PTB: significant psychological pressure, interrupted studies, and insufficient support from healthcare workers.

This study suggested heavy psychological pressure among college students with PTB. Carol AM and Yang et al. reported similar deficits in mental well being in TB patients [14,15]. The participants' psychological pressure were: anxiety before diagnosed with PTB, badly fear when diagnosed with PTB, anxiety in the beginning of treatment, excessive worry immediately before the first recheck, worry, expectancy and boredom during the whole treatment phase. Although the patients' emotions fluctuated as a whole, two key time points, the diagnosis of PTB and the first recheck approached after nearly one month treatment, manifested especially obvious.

Our finding supports the report that the first reaction to diagnosis of PTB is fear [8,9]. This is similar to Rajeswari's study, which reported the first reaction to diagnosis of tuberculosis was quite distressing, including worry, depression and suicidal thoughts for most patients [16]. However, the participants in Carlo's study expressed a wide range of emotions, as being calm, accepting or apathetic, scared or afraid, shocked or surprised, "devastated", worried or concerned, depressed[17]. The discrepancy between Carlo's report and this study may caused by the different sampling. For example, Carlo's study also recruited those TB patients suffered from other chronic diseases. Psychological reaction at the diagnosis point is the most intensive in the whole treatment. College students with PTB reported that their fear mainly derived from the traditional viewpoints that 'nine in ten who diagnosed with PTB will die' and 'PTB can not be cured'. These misconceptions still exist in China, and mislead the judgement of college students with PTB. Therefore, health workers should provide mental support for PTB patient on time, and it is also necessary to educate the college students that the 90% PTB patients can be cured by standard treatment.

One salient aspect of our findings was that college students with PTB were excessively worried immediately before the first recheck. This is another period when the participants' negative emotion manifested especially obvious during the whole treatment. The result does not consist with other quantitative studies which reported that negative emotions declined with treatment [14,16]. The difference may be caused by the discrepancy between the time points the quantitative studies selected and the time points our participants reported. The participants reported that this negative emotional derived from intensive concern on the result of first recheck. Intensive phase may be prolonged if there is little or no effect after one-month treatment. Thus, the whole treatment course will be prolonged. In this study, nearly all the participants expressed their strong desire of early recovery and they expected to return school as soon as possible. Therefore, result of first recheck was fully concerned by the participants, which leads to excessive worry.

In China, from 'one couple a child' policy, most college students are the only child in their family. Their parents and family members spoil them, which results in some of them being unable to live independently. At the same time, most of the Chinese college students go to colleges that are far away from their hometown. For most of them, it is the first time they left their parents, and they have to live independently. As a result, when they are suddenly diagnosed with PTB, they have to live in the hospital to receive a long-term treatment, and they are confused in dealing with it. A coping crisis occurs.

Therefore, healthcare workers should not only provide biotherapy to college students with PTB, but also offer psychological support for them according to the source of psychological pressure, especially at the time of being diagnosed with PTB and immediately before the first recheck.

For college students with PTB, disruption of study was the biggest problem caused by PTB. All of them were eager to study during the treatment. The Chinese government states clearly that if an infectious patient received regular and formal treatment for 2-3 weeks and the infectiousness can not be detected, then the patient can take part in normal work, study, and social activities; a non-infectious patient can take anti-TB drugs under directly supervision of healthcare workers and continue to study or work [18]. But in order to ensure that other students will not be infected, most of colleges state that freshman diagnosed with PTB during entering physical examination must be absence from school for one year to receive treatment. If the patients have already studied at the college, they must receive treatment at home or in the hospital. They must not go back to school until the continuation phase ended. The participants felt inconveniently, since they have to rent a room outside to keep studying. All the participants expressed intensively that they were unwilling to delay their studies and postpone graduation. They reported that the related school regulation brought heavy pressure to them. There is little international report on discontinuation of study among college students with PTB. Only one study in China reported that seniors suspending of school were disturbed on their job seeking opportunities, which left them anxious and depressed [19]. In order to ease the pressure of college student with PTB during the treatment, colleges should adjust their rules on the basis of the governmental guidelines on PTB.

Many studies reported that economic pressure was an important factor of delaying to health providers and not adherence to treatment among PTB patients [20-22]. However, the expenditure was not among the concerns of most participants in this study. 70~80% of the treatment fee could be reimbursed. More than a half of the participants reported the treatment fees were affordable. However, it was a heavy burden of participants from low-income families. In this study, participants who have been hospitalized paid about RMB5000 by themselves for the treatment, but the outpatients who received treatment under the TB free treatment policy only spent RMB1000 or so by themselves. Therefore, colleges should not directly send students diagnosed with PTB to TB hospital not considering the students' willing, since receiving treatment under TB free treatment policy could lower their expenditure greatly.

This study also found that family members could strongly support college students with PTB. However, little support from Healthcare workers, especially doctors and nurses in the college clinics and TB hospitals were received. Christopher and Amir et al. reported similarly that the healthcare workers did not help the patients with their concerns and problems during the treatment, patients were not satisfied with the care provided [23,24]. In London, specialist TB nurses at the TB clinic assess new TB patient's needs for treatment after they have started treatment as soon as possible [25]. But in China, most of the healthcare workers are busy with their works, for example, providing effective medical treatment for patients, and thus with little time to communicate with the patients or focus on what the patients concern about. Most of the participants were unknown of the TB free treatment policy which could lower their costs and other TB information. Healthcare workers should strengthen communication with college students with PTB, seek the needs of them and provide sufficient support for them.

This study has some limitations. First, a small and purposive sample means findings were not representative of college students with PTB in Shaanxi province. Secondly, there was no participant was ascertained with smear-positive, recurrent PTB, or multi-drug resistant PTB in this study. The experiences and psychological process of such patients may be different from participants revealed in this study. Thirdly, although we got touch with the participants through doctors in different college clinics, some of the inpatients were in the same TB hospital, even in the same ward and with close communication. Therefore, their viewpoints on the interview questions may be influenced by each other.

## Conclusions

This study explored the experiences and psychological process of college students with PTB before the onset of the illness and during the treatment phase, and the findings would be of great value for developing a comprehensive plan on psychological and social support for college students with PTB. Their psychological pressure was significant during the treatment. In addition, there was serious conflict between treatment and study during their treatment, social support provided for them was insufficient. Finally, doctors and nurses in college clinics and TB hospitals as well as TB control and prevention members should provide psychological supports for the college students with PTB according to the character of their psychological characteristics and offer social supports through strengthening deeper communication. Colleges should follow governmental policies on TB exactly and provide opportunities for the patients to continue their studies.

## Competing interests

The authors declare that they have no competing interests.

## Authors' contributions

SRZ conceived of the study, participated in design and coordination, performed the data collection, data analysis and draft the manuscript. HY supervised the study, helped to draft the manuscript and made critical revision to the paper. JJZ performed the data collection, data analysis, draft the manuscript and made critical revision to the paper. THZ and XHL performed data collection and analysis and helped to draft the manuscript. YPZ participated in the data collection, analysis and made critical revision to the paper. All authors read and approved the final manuscript.

## Appendix

### Appendix 1 - interview schedule

1. How are you getting on since your illness?

2. Will you please explain the possible causes of the disease from your side? (When and where were you infected possibly?)

3. Please talk about the process of your diagnosis

• In which way were you diagnosed? (Seeing a doctor or medical examination?)

• What are your symptoms?

• How long did it take for your diagnosis?

• which health facilities did you go to? With which health facilities are you diagnosed?

4. Would you please describe your feelings when suffered from the disease?

Is there anyone help you? How did he (them) help you?

5. What problems has your illness brought to you?

How did you deal with them?

6. Do you prefer to stay at home or school during the treatment period? Why?

7. What are you concerned about most?

8. Do you have any knowledge about PTB?

• Please tell me your knowledge on PTB.

• Did the doctor and nurse in your college clinic or the hospital tell you information on PTB?

• How do you access to information on PTB? From who and how would you like to get information on PTB?

9. Do you know the government's policy on TB?

• Do you know convergence management policy? What is it?

• Do you know TB free treatment policy? What is it?

• Who have ever told you the two policies?

• Do you have any suggestions on it?

## Pre-publication history

The pre-publication history for this paper can be accessed here:

http://www.biomedcentral.com/1471-2334/10/174/prepub
